# Contingency Management: Incentives for Sobriety

**Published:** 1999

**Authors:** Stephen T. Higgins, Nancy M. Petry

**Affiliations:** Stephen T. Higgins, Ph.D., is a professor in the Departments of Psychiatry and Psychology at the University of Vermont, Burlington, Vermont. Nancy M. Petry, Ph.D., is an assistant professor in the Department of Psychiatry at the University of Connecticut School of Medicine, Farmington, Connecticut

**Keywords:** addiction care, treatment method, intervention, reinforcement, treatment outcome, AOD (alcohol and other drug) abstinence, problematic AOD use, multiple drug use, AOD dependence, treatment goals, treatment research, patient compliance, employment, animal model, literature review

## Abstract

Contingency management (CM), the systematic reinforcement of desired behaviors and the withholding of reinforcement or punishment of undesired behaviors, is an effective strategy in the treatment of alcohol and other drug (AOD) use disorders. Animal research provides the conceptual basis for using CM in AOD abuse treatment, and human studies have demonstrated the effectiveness of CM interventions in reducing AOD use; improving treatment attendance; and reinforcing other treatment goals, such as complying with a medication regimen or obtaining employment.

Contingency management (CM)^1^ is a strategy used in alcohol and other drug (AOD) abuse treatment to encourage positive behavior change (e.g., abstinence) in patients by providing reinforcing consequences when patients meet treatment goals and by withholding those consequences or providing punitive measures when patients engage in the undesired behavior (e.g., drinking). For example, positive consequences for abstinence may include receipt of vouchers that are exchangeable for retail goods, whereas negative consequences for drinking may include withholding of vouchers or an unfavorable report to a parole officer. The reinforcing or punishing consequences may be contingent on objective evidence of recent AOD use or on another behavior important in the treatment process, such as compliance with a medication regimen or regular clinic attendance. Often, clinicians implement CM procedures through written contracts that detail the desired behavior change, duration of intervention, frequency of monitoring, and potential consequences of the patient’s success or failure in making the agreed-upon behavior changes.

An extensive body of research supports CM’s efficacy in treating various behavioral disorders, including AOD abuse (Higgins and Silverman 1999; Higgins et al. 1998). This article briefly reviews the conceptual background and empirical research demonstrating the efficacy of CM in AOD abuse treatment.

## Conceptual and Basic Science Foundations

The use of reinforcing and punishing consequences to alter the form and frequency of voluntary behavior is known as operant conditioning, a method that provides the conceptual framework for CM. Within the CM framework, AOD use is considered a form of operant behavior—that is, behavior that is maintained in part by the reinforcing biochemical effects of the abused substance and by reinforcing environmental influences (e.g., social reinforcement from peers).

Findings from animal research support the use of CM in the treatment of AOD use disorders. Such research demonstrates, for instance, that animals exhibit consumption patterns indicative of dependence and that researchers can modify animals’ AOD intake by using reinforcing and punishing consequences. Generally, laboratory animals voluntarily ingest the same substances that humans abuse (Griffiths et al. 1980). Rats and monkeys, for example, will voluntarily consume large quantities of cocaine, opioids, and alcohol. Neither a prior history of drug exposure nor physical dependence is necessary to support ongoing and stable patterns of AOD use in laboratory animals. Moreover, studies of voluntary AOD consumption by laboratory animals show that once a pattern of heavy consumption has been established, animals will complete cumbersome tasks (e.g., press a lever numerous times) to obtain and consume the desired substance. Additionally, laboratory animals will forgo other reinforcers, including sweet liquids, high-calorie solutions, and in some cases even basic sustenance, to engage in AOD use (Petry and Heyman 1995). These behavioral patterns are analogous to those exhibited by AOD-dependent humans, who often spend significant amounts of time and money abusing alcohol and recovering from AOD use, and who often give up recreation, employment, and family activities to do so.

These findings—that laboratory animals voluntarily consume many of the same substances that humans abuse and exhibit consumption patterns indicative of dependence—suggest that the necessary neurobiological systems to experience AOD-induced reinforcement and to engage in compulsive AOD use are widely represented across different species. However, laboratory studies also indicate that individual and environmental factors clearly influence susceptibility to AOD use and abuse (Wolffgramm and Heyne 1995). For example, when first exposed to alcohol, rats bred for high alcohol intake will voluntarily consume larger amounts of alcohol than non-selectively bred rats. However, even initially low-alcohol-consuming rats will voluntarily consume large quantities of alcohol under certain conditions. For example, stress, social isolation, and reduced access to food, liquid, or opportunities for exercise all promote AOD use in laboratory animals. Therefore, susceptibility to the reinforcing effects of AODs appears to be a product of normal neurobiological systems common to many species that can be heightened by certain individual and environmental factors.

Findings from animal studies may have implications for human AOD abuse treatment in that the environmental conditions which promote AOD use in laboratory animals seem similar to those associated with excessive AOD use in humans (Griffiths et al. 1980). Animal studies also demonstrate that conditions can be altered to reduce AOD use even after high levels of use have been established. Such studies suggest that an increase in both the availability of alternative, nondrug sources of reinforcement and the direct and indirect losses associated with AOD use are related to decreased AOD use (Higgins 1996). For example, providing food, liquids, or novel environmental alternatives reduces AOD use in animals, just as providing entertainment or financial alternatives reduces AOD use in humans. Although, as previously noted, animals and humans will work hard or forgo other reinforcers to consume addictive substances, an increase in the availability of alternative reinforcers will decrease AOD consumption. Furthermore, increasing the demands required to obtain the desired substance (e.g., increasing the number of times an animal must press a lever to obtain the alcohol) or directly associating substance use with the loss of other desired goods (e.g., withholding vouchers as a consequence of alcohol use) reduces AOD use as well (Higgins 1996).

CM interventions are based on the view that AOD use is a behavior that is influenced by neurobiological and environmental factors and that such behavior can be changed by applying consistent environmental consequences to reinforce the targeted behavior change. The following sections of this article describe research on the application of CM in the treatment of alcoholism and problem drinking as well as of other drug use disorders.

## Early Research and Application of CM in the Treatment of Problem Drinking

Research during the 1960s, 1970s, and 1980s examined the role of CM in alcoholism treatment as a strategy for reinforcing abstinence as well as accomplishing other treatment goals, including medication compliance and treatment attendance.

### Reinforcement of Abstinence

In an early study of CM in alcoholism treatment, Miller (1975) found that by providing tangible reinforcers to public inebriates, contingent on negative breath-alcohol tests, researchers could effectively reduce public inebriation. In the study, 20 public inebriates were randomly assigned to one of two groups: a contingent group or a noncontingent group. A person in the contingent group received shelter, employment, food, and clothing from local social services agencies only when he or she remained sober. Members of the noncontingent group received social services regardless of their drinking behavior. The researchers assessed sobriety by conducting random breath-alcohol tests and through staff observation. When the researchers detected alcohol use in a contingent group member, they immediately suspended his or her social services for 5 days.

During the 2-month treatment period, the contingent group’s arrest rate for public drunkenness decreased from an average of 1.7 arrests per 2 months to an average of 0.3 arrests per 2 months. Conversely, the noncontingent group’s arrest rate showed little change, decreasing from an average of 1.4 arrests per 2 months to an average of 1.3 arrests per 2 months. In addition, members of the contingent group increased their average number of hours of employment over the study period, an effect that was not found in the noncontingent group.

Additional studies of CM have also indicated positive findings. For example, researchers have reported positive outcomes using CM to reinforce abstinence among adolescent alcohol abusers (Brigham et al. 1981) and among alcohol-abusing schizophrenics (Peniston 1988).

### Reinforcement of Medication Compliance

In addition to the direct reinforcement of abstinence, CM also has been used to reinforce compliance with medications prescribed to reduce drinking, such as disulfiram (Antabuse^®^). Patients taking disulfiram have severe adverse reactions to alcohol and are therefore unlikely to drink when taking the medication. Liebson and colleagues (1978) randomly assigned 23 alcoholics who were receiving methadone for heroin dependence to either a contingent or control group. In the contingent group, each patient’s continued methadone treatment was contingent on the patient’s ingestion of disulfiram. Patients who did not take the mandatory doses of disulfiram had their daily dose of methadone reduced until it reached zero and they were discontinued from the program or until they began or resumed taking disulfiram. In the control group, disulfiram therapy was recommended, but non-compliance had no effect on continued methadone treatment.

During the 6-month treatment period, patients in the contingent group drank, on average, on only 2 percent of the total number of days, compared with an average of 21 percent for the control group. In addition, a controlled case study suggested that a CM intervention involving supervised disulfiram ingestion was effective in reducing absenteeism among employees referred for drinking on the job (Robichaud et al. 1979).

Another study of similar CM procedures conducted with public drunkenness offenders found no positive effect. Gallant and colleagues (1968*a*) randomly assigned 84 repeat public drunkenness offenders to one of four 6-month treatments: (1) mandated group therapy, (2) mandated group therapy plus thrice weekly observed disulfiram ingestion, (3) thrice weekly observed disulfiram ingestion only, and (4) the usual sentence as well as an informal suggestion to attend an alcoholism clinic. In the first three groups, noncompliance was intended to result in an automatic 60-day sentence. Contingencies were not consistently applied, however, and no differences were noted between the groups. This lack of effect may be related, in part, to the fact that fewer than 10 percent of the study participants were available for the 6-month posttreatment evaluation.

### Reinforcement of Treatment Attendance

CM also has been used in alcoholism treatment to encourage patients to attend treatment sessions. Gallant and colleagues (1968*b*) found beneficial effects in making treatment attendance a condition of parole among alcoholics recently released from 1-year or longer sentences for major alcohol-related offenses. In the study, the researchers randomly assigned 19 alcoholics to either outpatient treatment at which attendance was a parole requirement or outpatient treatment at which attendance after the first appointment was urged but not required. For parolees in the first group, failure to attend treatment resulted in a return to prison to serve the remainder of their sentence. Ninety percent of this group attended treatment regularly for 6 months, compared with only 11 percent of the voluntary treatment group. After 1 year, 70 percent of the contingent group was abstinent and working, whereas 78 percent of the voluntary treatment group was either in prison or had violated parole.

In another application of CM in the criminal justice system, Ersner-Hershfield and colleagues (1981) evaluated the use of contingencies to promote treatment attendance among offenders convicted of driving under the influence (DUI). Sixty-seven DUI offenders were randomly assigned to either a program based on behavioral self-control or a program consisting of alcohol education, relaxation training, and guided re-evaluations of situations associated with drinking and driving. One-half of the offenders in each group participated in a deposit system in which they paid $50 at the first session and received a $5 reimbursement check for each subsequent session that they attended. The two treatments were equally effective; however, participants in the refundable-deposit groups had fewer unexcused absences than patients in the no-deposit groups.

As described above, several studies have demonstrated the efficacy of CM in reducing drinking and increasing treatment compliance among alcoholics and problem drinkers. Studies that did not report positive effects were hindered by inconsistent monitoring and application of consequences, which may account for the poor outcomes (Gallant 1968*a*). Until recently, research on the use of CM to treat problem drinking ceased, whereas CM research flourished in the area of illicit and polydrug abuse treatment. Those developments are described below.

### Expansion and Further Development of CM Procedures

Recent CM interventions have been structured around four central principles. First, the clinician arranges for regular testing to ensure that the patient’s use of the targeted substance is readily detected. Second, the clinician provides agreed-upon tangible reinforcers when abstinence is demonstrated. Third, the clinician withholds the designated incentives from the patient when substance use is detected. Fourth, the clinician assists the patient in establishing alternate and healthier activities (e.g., a better paying job, improved family relations, enjoyable social and recreational activities) to compete with the reinforcement derived from the AOD-abusing lifestyle. CM is usually, but not always, included as part of a comprehensive treatment plan involving other psychosocial and pharmacological interventions.

## CM in Illicit and Polydrug Abuse Treatment

Clinicians have used CM in illicit drug abuse treatment to reinforce abstinence as well as other treatment-related behaviors, such as treatment attendance or compliance with a medication regimen.

### Reinforcement of Abstinence

In many CM interventions involving illicit drug abusers, clients submit urine specimens several times weekly to be screened for evidence of drug use. When the specimens are negative for drug use, clients receive reinforcers, such as take-home doses of methadone, increases in clinic privileges, money, and vouchers exchangeable for retail goods. In many of the CM studies that use vouchers as reinforcers, the value of the earned vouchers escalates as the patient demonstrates consecutively longer periods of abstinence. Submission of samples showing drug use results in no reinforcer and sometimes a punishment (i.e., the voucher amount decreased to a lower value or loss of take-home privileges).

Research indicates that AOD abuse treatments incorporating CM are more effective than standard case management, 12-step-oriented counseling, and behavior therapies delivered without a CM component (Higgins and Silverman 1999; Petry et al. in press). CM interventions generally retain patients in treatment longer and reduce AOD use more than do comparison treatments or other comparison conditions. In a study with 38 cocaine-dependent adults, for example, researchers randomly assigned patients to 24 weeks of community reinforcement approach (CRA) therapy, an operant-based treatment methodology originally developed to treat chronic alcoholics (see the article in this issue by Miller et al., pp. 116–121, for more details), as well as CM or 12-step-oriented counseling (Higgins et al. 1993). In the CM condition, patients received vouchers for submitting cocaine-negative urine specimens. Fifty-eight percent of patients in the CM condition remained in treatment throughout the study, compared with 11 percent of patients in the comparison group. CRA plus CM was effective in reducing cocaine use as well. Sixty-eight percent of clients in the CM condition maintained at least 8 weeks of continuous cocaine abstinence, compared with 11 percent of patients in the comparison group.

In the aforementioned study, two aspects of treatment varied between groups: provision of contingencies and orientation of therapy. A subsequent study demonstrated the contribution of CM to the beneficial effects observed (Higgins et al. 1994). Again, researchers randomly assigned cocaine-dependent adults to one of two conditions: CRA therapy plus CM using vouchers or CRA therapy alone. Seventy-five percent of the patients who received CRA plus CM completed the 24-week study, compared with 40 percent of the patients who only received CRA therapy. Fifty-five percent of the patients in the CM group achieved at least 2 months of continuous cocaine abstinence, compared with 15 percent of the comparison group. Based on these findings and similar results from other studies, the National Institute on Drug Abuse published a therapy manual detailing how to implement this treatment with cocaine-dependent outpatients, including the sizable subgroup who are also alcohol dependent (Budney and Higgins 1998).

Silverman and colleagues (1996) further investigated the use of a voucher-based incentive program among inner-city intravenous cocaine abusers. The researchers randomly assigned cocaine-abusing methadone patients either to receive vouchers contingent on submission of cocaine-free urine specimens (i.e., contingent group) or to receive vouchers regardless of drug test results (i.e., noncontingent group). Members of the noncontingent group received, on average, the same overall number of vouchers as patients in the contingent group, and both groups were retained in treatment for similar durations. Forty-seven percent of the clients in the contingent group tested negative for cocaine for 6 or more consecutive weeks, compared with only 6 percent of those in the noncontingent group, thereby further demonstrating the utility of CM for reducing cocaine use even in this difficult-to-treat population. Other randomized, controlled studies with opioid-dependent patients have shown that providing money, voucher incentives, or clinic privileges contingent on objective indicators of drug abstinence can reduce illicit drug use (Higgins et al. 1998).

### Reinforcing Other Treatment-Related Goals

A few recent studies among illicit drug users have evaluated the use of CM procedures to reinforce not only abstinence but also other treatment goals (Bickel et al. 1997; Iguchi et al. 1997). For example, in a study of opioid-dependent clients, therapists used vouchers to reinforce beneficial, non-drug-related activities in addition to abstinence (Bickel et al. 1997). Clients chose three treatment-related activities that they aimed to complete each week. Such activities included attending a medical appointment if the goal was to improve health, taking their child to the library if the goal was to improve parenting, or applying for a job if the goal was to obtain employment. Patients received vouchers when they presented documentation verifying that they had completed a designated activity. Patients who completed their chosen activities were more likely to remain abstinent than those who did not meet their activity goals. Reinforcing compliance with treatment-related activities may encourage patients to acquire new skills and overcome psychosocial difficulties associated with their AOD abuse. This approach is somewhat analogous to CRA.

## CM in the Treatment of Problem Drinking and Alcoholism

After a relatively long hiatus, researchers have again begun studying the use of CM in alcoholism treatment, partly as an outgrowth of the recent research demonstrating its efficacy with illicit and polydrug abusers.

In a recent CM study, 42 alcohol-dependent patients entering an intensive outpatient substance abuse clinic received either standard treatment plus CM or standard treatment only (Petry et al. in press). Standard treatment consisted of daily 5-hour group sessions focusing on relapse prevention, social and recreational training (i.e., planning alternative evening activities), 12-step-oriented groups, and AIDS education. This treatment continued for 4 weeks, after which patients were transferred to aftercare. The after-care component consisted of similar group sessions held 1 to 3 days per week. Patients in both groups (i.e., standard treatment plus CM or standard treatment only) provided breath samples to test for alcohol use. These breath samples were submitted daily for the first 4 weeks of treatment and then weekly during the aftercare period. Patients in the CM group earned the chance to win a prize for each negative breath sample they submitted and for each of three preset activities that they completed during the week. The prizes ranged in value from $1 to $100. Eighty-four percent of the patients in the CM group remained in treatment for the entire 8-week period, compared with only 22 percent of the patients in the standard treatment group. By the end of the treatment period, 69 percent of the patients in the contingent group had not yet experienced a relapse to alcohol use, compared with 39 percent of the patients in the standard treatment group. These results suggest that this CM procedure, which reinforced both abstinence and compliance with other treatment goals, was effective in retaining alcohol-dependent patients in treatment and reducing relapse.

Researchers are currently developing a project specifically designed to simultaneously reinforce abstinence and provide skills development in patients (Silverman, K., personal communication, January 1999). Chronically unemployed, homeless alcoholics will be randomly assigned to one of three groups. Members of the first group will receive data entry training and paid employment, but they will only be allowed to work and earn vouchers when they abstain from alcohol use. The second group will receive data entry training and employment regardless of their alcohol use. The third group will not receive either training or employment but will receive vouchers regardless of alcohol use. This study will evaluate CM’s efficacy in improving psychosocial functioning and in reducing alcohol use among one of the most difficult-to-treat populations.

## Conclusions and Future Directions

CM interventions among alcoholics, problem drinkers, and illicit-drug abusers have been found to be effective in reducing AOD use; retaining patients in treatment; improving medication compliance; and promoting participation in other treatment-related goals, such as employment. The reinforcers used in these interventions have included special privileges, money, methadone doses, vouchers, and prizes. Studies of CM across various patient populations that target various behaviors and use various reinforcers have found that CM generally improves outcomes relative to comparison treatments. Although CM is generally effective, proper implementation is important. In particular, treatment staff should monitor patients frequently and provide reinforcers consistently. Future research may need to focus on staff training to increase the consistency with which reinforcers are applied, especially as CM is used in non-research-based settings.

The use of CM in alcoholism treatment may be limited, however, by the technology available to test for alcohol use. Most CM interventions targeting drug abstinence screen patients’ urine specimens several times weekly. Because most drug-testing procedures can detect drug use over a 2- to 3-day period, twice or thrice weekly monitoring can detect any drug use throughout the week. However, alcohol testing is less sensitive. Breath alcohol tests can only determine whether a person has consumed alcohol over the past 4 to 12 hours; thus, the submission of negative samples confirms abstinence for only a relatively brief time period. Alcohol urine tests cannot detect use over a much longer time period than can breath tests, and blood alcohol tests are more invasive and still fail to extend the time period over which alcohol use can be detected. Researchers are evaluating the use of biological markers, which reflect the physiological changes that occur in the body after alcohol use, to detect both heavy and recent drinking even after the breath alcohol level reaches zero (Allen and Litten 1998). The tests for such biological markers require the shipment of specimens to a laboratory for analysis and therefore cannot provide immediate feedback, which is possible with some tests for illegal drugs. Thus, researchers face some fundamental problems when designing CM interventions to reduce drinking. As more studies evaluate CM among alcohol-dependent populations, researchers will need to consider these technological difficulties.

In addition to the goal of abstinence, CM interventions also are being used among alcohol-dependent clients to reinforce other treatment-related goals, such as treatment attendance and employment. This practice may present a more comprehensive approach to treating AOD use disorders and may improve patients’ psychosocial problems. Future research is needed to further evaluate the efficacy of these and related procedures.

Followup studies on the efficacy of CM have demonstrated beneficial long-term effects but have also found evidence of relapse in about the same proportion as is seen with other psychological treatments for AOD abuse disorders (Higgins et al. 1995). CM studies with larger numbers of participants are needed to more carefully quantify relapse rates with this treatment approach. Further research is also needed to evaluate the optimal length of treatment with CM and the use of reinforcers to improve longer term outcomes. For example, treatment gains may be maintained by improving the transition from the use of more contrived reinforcers (e.g., vouchers) to more naturally occurring reinforcers (e.g., obtaining and maintaining employment) as well as by altering reinforcement schedules from continuous reinforcement during initial treatment to more variable reinforcement schedules as the treatment progresses.

Finally, CM may be well suited for treating a variety of populations. Positive reinforcement procedures may effectively prevent alcohol use in high-risk adolescents and pre-adolescents. Additionally, these procedures may help encourage non-treatment-seeking patients to begin alcoholism treatment. CM is not a “magic bullet” for treating any group, however, and researchers must design and implement CM interventions carefully to ensure their effectiveness. Nevertheless, these procedures offer the opportunity for clinicians to effectively manage and treat some of the most challenging problems and populations in the field of AOD abuse.

## Footnotes

1 Editor’s Note: Community-reinforcement approach (CRA), a treatment methodology that uses strategies similar to those used in CM, is discussed in a separate article in this issue by Miller et al., pp. 116–121.
